# Genome-scale characterization of RNA tertiary structures and their functional impact by RNA solvent accessibility prediction

**DOI:** 10.1261/rna.057364.116

**Published:** 2017-01

**Authors:** Yuedong Yang, Xiaomei Li, Huiying Zhao, Jian Zhan, Jihua Wang, Yaoqi Zhou

**Affiliations:** 1 Institute for Glycomics and School of Information and Communication Technology, Griffith University, Gold Coast, QLD 4222, Australia; 2 School of Computer Science and Information Engineering, Hefei University of Technology, Hefei 230009, China; 3 Institute of Health and Biomedical Innovation, Queensland University of Technology, Queensland 4222, Australia; 4 Shandong Provincial Key Laboratory of Biophysics, Institute of Biophysics, Dezhou University, Dezhou 253023, China

**Keywords:** RNA structure prediction, in vivo RNA structure, solvent accessibility, genetic variants, conformational selections, induced fit, single nucleotide variants, 1000 Genomes Project

## Abstract

As most RNA structures are elusive to structure determination, obtaining solvent accessible surface areas (ASAs) of nucleotides in an RNA structure is an important first step to characterize potential functional sites and core structural regions. Here, we developed RNAsnap, the first machine-learning method trained on protein-bound RNA structures for solvent accessibility prediction. Built on sequence profiles from multiple sequence alignment (RNAsnap-prof), the method provided robust prediction in fivefold cross-validation and an independent test (Pearson correlation coefficients, *r*, between predicted and actual ASA values are 0.66 and 0.63, respectively). Application of the method to 6178 mRNAs revealed its positive correlation to mRNA accessibility by dimethyl sulphate (DMS) experimentally measured in vivo (*r* = 0.37) but not in vitro (*r* = 0.07), despite the lack of training on mRNAs and the fact that DMS accessibility is only an approximation to solvent accessibility. We further found strong association across coding and noncoding regions between predicted solvent accessibility of the mutation site of a single nucleotide variant (SNV) and the frequency of that variant in the population for 2.2 million SNVs obtained in the 1000 Genomes Project. Moreover, mapping solvent accessibility of RNAs to the human genome indicated that introns, 5′ cap of 5′ and 3′ cap of 3′ untranslated regions, are more solvent accessible, consistent with their respective functional roles. These results support conformational selections as the mechanism for the formation of RNA–protein complexes and highlight the utility of genome-scale characterization of RNA tertiary structures by RNAsnap. The server and its stand-alone downloadable version are available at http://sparks-lab.org.

## INTRODUCTION

RNA plays pivotal roles in essentially every process in living cells, acting as gene regulators, molecular recognizers, temperature sensors, and reaction catalyzers (Sharp 2009). Similar to proteins, RNA performs this wide variety of functions by folding into diverse base-pairing patterns (secondary) and three-dimensional (tertiary) structures (Wan et al. 2011; Mortimer et al. 2014). The flexible nature of RNA, however, makes it difficult to determine RNA tertiary structures experimentally by either X-ray crystallography or nuclear magnetic resonance (NMR) spectroscopy. As of May 1, 2016, only 3155 RNA-containing structures were available in the Protein Data Bank (PDB) (Rose et al. 2015).

The difficulty in RNA structure determination leads to development of chemical probes combined with next-generation sequencing for genome-wide measurement of RNA secondary structure and solvent accessibility of RNA bases in its tertiary structure (Westhof and Romby 2010; Doerr 2014; Kwok et al. 2015). Such genome-scale structural information significantly advances our understanding of RNA structure–function relations (Wan et al. 2011; Mortimer et al. 2014), because RNA secondary structure and accessibility can be explored in vivo. In contrast, RNA structures determined by in vitro methods such as NMR and X-ray crystallography may or may not reflect the functional state of RNA in cells (Blount and Uhlenbeck 2005; Yajima et al. 2007). Indeed, it has been shown that there is a large difference between in vivo and in vitro RNA structures (Zaug and Cech 1995; Rouskin et al. 2014).

Time-consuming and costly experimental studies make it necessary to predict RNA structures computationally. Progress has been made in predicting RNA tertiary structures directly from RNA sequences (Seetin and Mathews 2012; Puton et al. 2014; Xu and Chen 2015). It is still difficult, however, to predict noncanonical pairs, such as A–A and G–A, (Miao et al. 2015), which are essential for correctly folded tertiary structures (Tinoco and Bustamante 1999). Except for template-based homology modeling, the methods for ab initio RNA structure prediction (Seetin and Mathews 2012; Puton et al. 2014; Xu and Chen 2015) were built to predict the structures of isolated RNA chains that may or may not reflect their functional conformations. As a result, commonly used computational techniques for probing RNA structures are limited to the secondary structure level. The accuracy of secondary structure prediction is at 50%–75% in the absence of experimental restraints (Seetin and Mathews 2012; Hajdin et al. 2013).

De novo prediction of RNA secondary and tertiary structures is challenging. This is because RNA primary structures (sequences) have only four hydrophilic side chains in two sizes (purine and pyrimidine). RNA tertiary structural folding is further complicated by degenerate base-pairing and stacking and stable RNA duplexes that result in an ensemble of energetically similar structural states for most RNAs (Herschlag 1995; Woodson 2010; Dethoff et al. 2012; Herschlag et al. 2015). Functional structures of RNAs often result from induced fit (Williamson 2000; Leulliot and Varani 2001) or conformational selection (selected by interacting partners from a dynamic ensemble of preexisting structures) (Fürtig et al. 2010; Herschlag et al. 2015).

Structural ensembles for isolated RNA chains raise a practically and fundamentally important question: Are functional tertiary structures predictable in the absence of their binding partners? To address this question, we focus on the solvent accessibility or the fraction of solvent accessible surface area (ASA) of nucleotides in the tertiary structure of an RNA chain. RNA solvent accessibility, reflecting the level of exposure of a base to solvent, is a one-dimensional structural property of tertiary structure that is more amenable for computational prediction than three-dimensional structure, as demonstrated in predicting the solvent accessibility of proteins (Zhou and Faraggi 2010; Heffernan et al. 2015). The ability to predict RNA accessibility of functional tertiary structures directly from RNA sequences would support the notion that functional RNA structures are encoded in their nucleotide sequences alone (i.e., without inputting information from their binding partners).

Here, we built the first machine-learning model on protein-bound RNA structures for RNA solvent accessibility prediction (named as RNAsnap). We showed that the model yields a robust performance in cross-validation and independent tests. Its genome-scale application to mRNA indicates a positive correlation to the in vivo genome-scale measured RNA reactivity to dimethyl sulphate (DMS) (Rouskin et al. 2014). Moreover, a smaller predicted ASA of the mutation site (more buried site) of a single-nucleotide variant is associated with lower frequency of that variant (minor allele frequency) found in the 1000 Genomes Project (Huang et al. 2012), indicating detrimental effects of disrupting inaccessible RNA core structures. These results suggest that solvent accessibility of functional RNA structures can be captured with reasonable accuracy without knowing their interacting partners. That is, conformational selection is the dominant mechanism for folding functional RNA structures. RNAsnap provides the first powerful tool for genome-scale characterization of RNA tertiary structure.

## RESULTS

**FIGURE 1.**
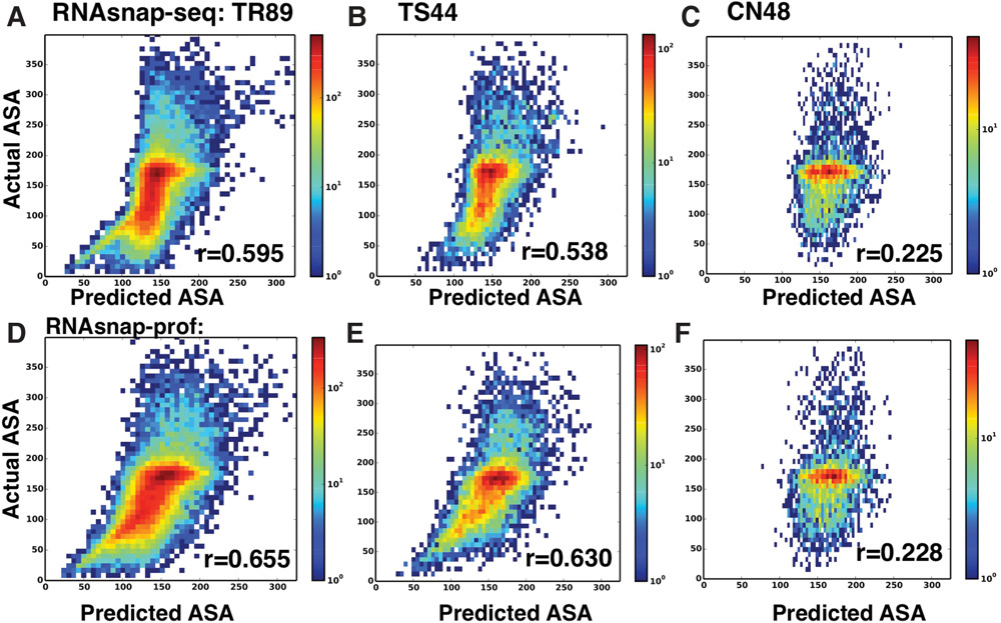
Performance on the training (TR89) and the independent test (TS44) sets of protein-bound structures and the control set of protein-free structures (CN48) by RNAsnap. The density plot of predicted versus actual ASA on TR89 (*A*), TS44 (*B*), and CN48 (*C*) by RNAsnap-seq and on TR89 (*D*), TS44 (*E*), and CN48 (*F*) by RNAsnap-prof. PCC values (*r*) are as labeled.

Using 89 high-resolution (<3.0 Å), nonredundant RNA structures in complex with proteins for training (TR89), we developed two separate support-vector-machine (SVM) models by inputting either the query RNA sequence alone or the sequence profile from multiple sequence alignment against the query sequence. These two models are called RNAsnap-seq and RNAsnap-prof, respectively. Figure 1 compares predicted with actual solvent accessibility in a density plot. We measured the accuracy by the Pearson's correlation coefficient (PCC) between predicted and actual solvent accessibility derived from RNA structures. RNAsnap-seq achieves 0.595 for fivefold cross-validation in the training TR89 set and 0.538 for the independent test set (TS44), but only 0.225 for 48 protein-free RNA structures (CN48). The corresponding PCC values for RNAsnap-prof are 0.655, 0.630, and 0.228, respectively. Similar accuracies between the cross-validation by TR89 and the independent test by TS44 confirm the robustness and generality of the SVM models developed. A slightly worse performance for the test set is likely because structural resolution of the structures in the test set (3.0–4.0 Å) is lower than those in the training set (<3.0 Å). We used lower resolution structures for testing because the number of nonredundant high-resolution structures is not large enough to set some structures aside for the independent test. We would like to note that all correlations observed here for TR89 and TS44 sets are statistically significant with a near-zero *P*-value (<10^−99^).

To examine whether the strong correlations (>0.6) found in the cross-validation and independent test set are biased toward specific nucleotides, we have calculated correlations for four bases, separately. For the fivefold cross-validation, the PCC values of RNAsnap-prof are 0.663 for A, 0.670 for C, 0.656 for G, and 0.626 for U, respectively. For the test set, the PCC values of RNAsnap-prof are 0.623 for A, 0.621 for C, 0.622 for G, and 0.656 for U, respectively. The results between predicted and actual ASAs for individual bases (A, C, G, U) for the test set are shown in Supplemental Figure S1. The order of PCC values for four bases seems to be random, and follows neither the sizes of nucleotides where adenine (A) and guanine (G) are larger than cytosine (C) and uracil (U), nor the abundance of A, C, G, and U in our data sets (A = 6516, C = 6743, G = 8734, U = 5223 in TR89, and A = 2253, C = 1951, G = 2270 and U = 1918 in TS44, respectively). In addition, the averages and standard deviations of actual ASA values of A, C, G, and U are 137 ± 59, 140 ± 47, 144 ± 48, and 145 ± 56, respectively, indicating small differences among them.

We investigated if there is a difference between protein-binding and nonbinding nucleotides. In our training and independent test, these two types of bases were not distinguished from each other. We found that if we remove the bases involved in binding to proteins in evaluation of correlation coefficients, we obtained a higher PCC (0.687 vs. 0.655) between predicted and actual ASA for TR89 by RNAsnap-prof. Thus, RNAsnap-prof can recognize exposed, nonbinding RNA bases as well as binding RNA bases.

**FIGURE 2.**
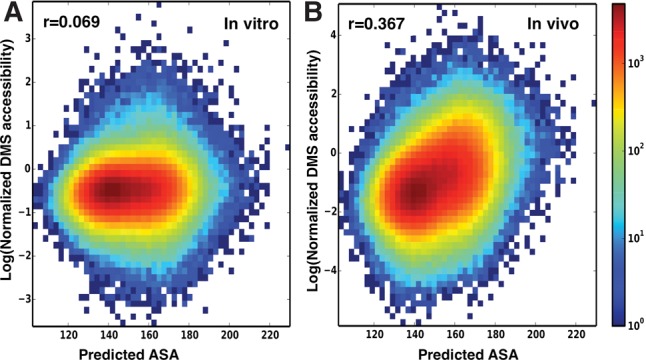
RNAsnap-prof prediction correlates to in vivo but not to in vitro data. The density plot of normalized in vitro (*A*) and in vivo (*B*) DMS accessibilities versus predicted ASA values by RNAsnap-prof. PCC values (*r*) are as labeled.

To further test RNAsnap, we obtained genome-wide accessibility data of mRNAs generated by utilizing DMS to detect unpaired and exposed adenine and cytosine residues (Rouskin et al. 2014). We noted that DMS reactivity was used to approximate solvent accessibility (Rouskin et al. 2014), which is calculated regardless of RNA paired or not. Nevertheless, it is of interest to examine if there exist some correlations. Figure 2 compares predicted ASAs by RNAsnap-prof with normalized DMS accessibility in vitro (Fig. 2A) and in vivo (Fig. 2B) for 6178 mRNAs from a human bone marrow K562 cell. PCCs are 0.069 and 0.367 between predicted solvent accessibility and DMS accessibility measured in vitro and in vivo, respectively. The correlation coefficient of predicted ASA to the in vivo data increases slightly to 0.370 if the calculations are limited to bases A and C that are reactive to DMS. Again, the correlations found here are statistically significant with a near-zero *P*-value (<10^−99^) given the fact that a large number of mRNAs (>6000) is involved.

As shown in Supplemental Figure S2A,B, RNAsnap-seq provides a similar performance with PCC = 0.085 to in vitro and PCC = 0.324 to in vivo DMS accessibility, respectively. This indicates that the correlation obtained is robust and not solely caused by RNA conservation information contained in the sequence profile used by RNAsnap-prof. As a comparison, we found that predicted secondary structure (unpaired probability) by RNAplfold (Bernhart et al. 2006) does not have a strong correlation to either in vitro (PCC = 0.024) or in vivo (PCC = 0.002) DMS accessibility (Supplemental Fig. S3A,B), highlighting the difference between predicted RNA secondary structure and predicted ASA in this study.

To further demonstrate that predicted ASA values reflect structural properties of functional conformations, we examined the relation between minor allele frequencies (MAF) of genetic variants and predicted ASA at mutation sites of the variants found in the 1000 Genomes Project based on healthy individuals (Huang et al. 2012). Allele frequencies result from complex interactions between human beings and their environment: Rare alleles are both population-specific and of functional significance (Marth et al. 2011), whereas frequent alleles (for late-onset diseases, in particular) are not necessarily benign. That is, the fitness of the allele with respect to its associated biological function is likely an underlying trend that appears only after averaging (removing noise from other factors). In previous studies (Hu and Ng 2012; Zhao et al. 2013), the expectation that highly populated genetic variants on average should be less likely to be associated with diseases was used successfully as an independent support for predicted disease susceptibility of genetic variants. Thus, if mutation-induced disruption of functional RNA structures is one of the potential causes of diseases (Halvorsen et al. 2010), we would expect that predicted RNA accessibilities at mutation sites positively correlate with average MAF values, similar to predicted protein solvent accessibility (Zhao et al. 2013).

**FIGURE 3.**
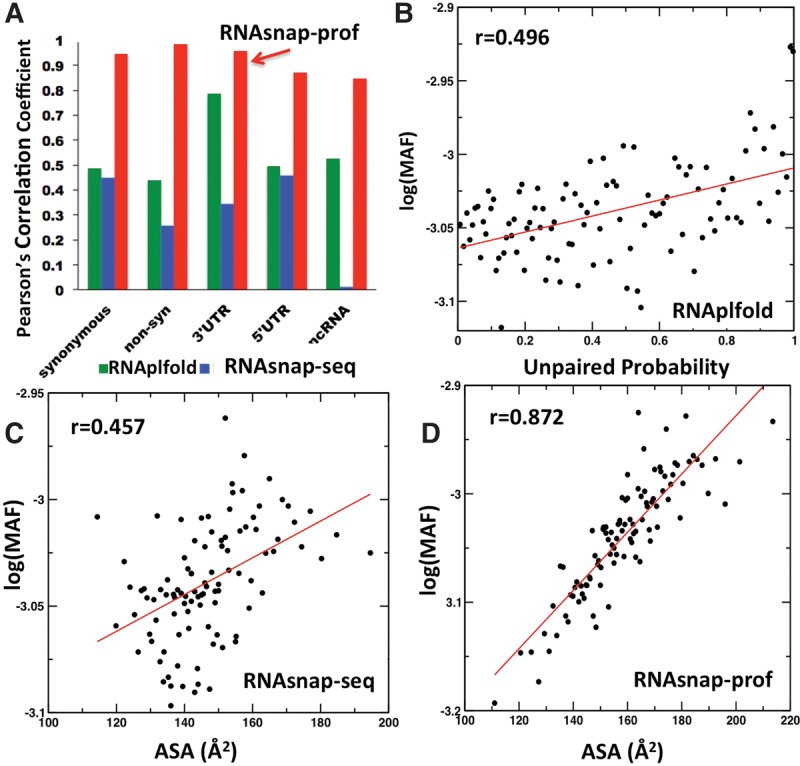
Positive association between minor allele frequencies (MAF) of genetic variants and predicted ASA or secondary structures at mutation sites. (*A*) Pearson's correlation coefficients between the average MAF of single-nucleotide variations and unpaired probabilities by RNAplfold, ASA by RNAsnap-seq, and ASA by RNAsnap-prof for synonymous and nonsynonymous mutations at the coding region, at 3′ and 5′ untranslated regions, and noncoding RNAs, respectively. For 5′ UTRs, the relation between the average MAF from the 1000 Genomes Project and the unpaired probability by RNAplfold, predicted ASA by RNAsnap-seq, and by RNAsnap-prof are shown in *B, C*, and *D*, respectively. The average was over bins sorted by predicted values. PCC values (*r*) are as labeled.

Figure 3A shows Pearson's correlation coefficients across different regions (synonymous and nonsynonymous mutations in coding regions, 3′ UTR, 5′ UTR, and ncRNA). As an example, the scatter plots for 5′ UTR are shown in Figure 3B–D for secondary structure by RNAplfold, ASA by RNAsnap-seq, and ASA by RNAsnap-prof, respectively (scatter plots for other regions can be found in Supplemental Figs. S4–S7). Unpaired probability in secondary structure at the mutation site shows positive correlations in all cases (i.e., less secondary structure at the mutation site corresponds to higher MAF on average), consistent with the deleterious functional impact from disruption of RNA secondary structure (Halvorsen et al. 2010; Salari et al. 2013). However, the strongest correlations are given by RNAsnap-prof-predicted ASA with PCC = 0.946, 0.984, 0.959, 0.872, and 0.846 for single nucleotide variants in synonymous and nonsynonymous mutations, 3′ UTR, 5′ UTR, and noncoding RNA, respectively. This reveals the trend that low MAF is associated with mutations occurring in buried (low accessibility) as well as in functional RNA regions because of sequence conservation information used in RNAsnap-prof. More can be found in the Discussion.

There are two types of single nucleotide variations: transversion between two-ring purine and one-ring pyrimidine (G↔T; A↔C; G↔C; A↔T); and transition between pyrimidines (T↔C) or purines (A↔G). We found that the correlations found above are slightly stronger for transition SNVs than for transversion SNVs. For example, for SNVs in 5′ UTR, PCC values between predicted ASA at the mutation site and the allele frequency of the mutation are 0.72 for 304 T→A,G and C→A,G transversions, 0.71 for 306 A→T,C and G→T,C transversions, and 0.83 for 983 T↔C and A↔G transitions. Thus, strong correlations are observed for all possible mutation types, indicating the consistent functional impact of mutating inaccessible RNA bases.

**FIGURE 4.**
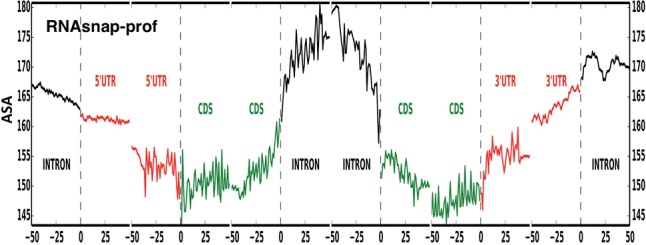
The position-dependent, average solvent accessibility pattern predicted by RNAsnap-prof reveals more exposed intron and 5′ cap of 5′ UTR and 3′ cap of 3′ UTR. The average is over 15,642 human gene transcripts containing 5′ UTR, exons, introns, and 3′ UTR of 100 bases or more.

To further illustrate the utility of predicted RNA accessibility, we performed genome-wide prediction of 15,642 human gene transcripts containing 5′ UTR, CDS, introns, and 3′ UTR of 100 bases or more. Figure 4 shows the average ASA of these transcripts near various boundaries predicted by RNAsnap-prof. Introns are more exposed, followed by a 5′ cap of 5′ UTR and a 3′ cap of 3′ UTR, whereas CDS and their surrounding 5′ and 3′ UTRs are more buried.

## DISCUSSION

This paper establishes the first machine-learning model trained on protein-bound RNA structures for predicting solvent accessibility. We found that a window size of 40 bases provided the best performance, as shown in Supplemental Figure S8. Ideally, the window size should cover the entire sequence of an RNA chain so that a machine-learning method can learn potential interactions between all nucleotides (local or nonlocal interactions). However, if the window size is too large, the large number of features will increase the risk of overtraining. This is why the window size needs to be optimized to maximize the performance while reducing the risk of overtraining by cross-validation and independent test. Our optimized window size is 40 because there are seven times more nucleotides (23,928) in large (>80) RNAs than those in small RNAs (3288). Covering the entire sequence for small RNAs by an optimized window size of 40 ensures the best learning for small RNAs while optimizing the performance for large RNAs.

This window size of 40 is much larger than a typical window size (8–10) of a protein used for predicting protein secondary structure and ASA (Zhou and Faraggi 2010; Heffernan et al. 2015). This is because 20 different amino acids of proteins with a typical window size of eight can generate 340 features [20×(2 × 8 + 1)], whereas four-letter-coded RNAs require us to expand the window size to 40 in order to achieve a similar number of features [324 = 4×(2 × 40 + 1)] so that a similar amount of information content can be learned from RNAs or from proteins. In other words, lower information content in RNA sequences than in protein sequences leads to the large window size for ASA prediction of RNA.

The resulting sequence-profile-based model (RNAsnap-prof) has achieved a correlation coefficient of 0.655 for fivefold cross-validation using TR89 and 0.630 for an independent test using TS44. The slightly poorer performance on the independent test set is somewhat expected because TS44 is made of lower resolution protein-bound structures (3.0 Å–4.0 Å), as TR89 has covered all nonredundant high-resolution structures (0–3.0 Å) in the Protein Data Bank. The consistent performance in TR89 and TS44 suggests that the solvent accessibility predictor developed here is robust.

Interestingly, both sequence- and profile-based models do not perform well on protein-free RNA structures (CN48), with overall PCC values between predicted and actual ASAs for the entire data set at about 0.2, compared with 0.5–0.6 for training and test sets of protein-bound structures. The reason for the failure of RNAsnap for protein-free structures is not clear. One possibility is that protein-free structures have a sequence–structure relation different from protein-bound structures. We attempted to develop a method specifically for protein-free RNA structures using CN48. We failed to produce a method that can achieve a reasonably accurate cross-validation result. This failure may be caused by the fact that CN48 has only 3858 bases, compared with >20,000 bases in TR89. More protein-free structures are needed to understand if solvent accessibility of protein-free RNA structures is as predictable as protein-bound structures from RNA sequences by machine-learning models. The inherently different structures between small and large RNAs are consistent with a recent finding that large-size RNA structures (>110 nt) behave in a manner more akin to unorganized collapse, whereas small RNAs and proteins fold with better packing (Liu and Hyeon 2016).

The robust prediction of protein-bound tertiary structural accessibility indicates for the first time that functional structures of RNA sequences are predictable in the absence of their binding partners. This is further supported by positive correlation between predicted ASA and measured in vivo DMS accessibility (PCC = 0.37) but not between predicted ASA and in vitro DMS accessibility (PCC = 0.07) for 6178 mRNAs. Such a positive correlation is obtained although there are only two mRNA fragments (38 and 48 bases, respectively) in our training set and the DMS reacts with both unpaired and exposed adenine/cytosine residues, rather than exposed residues only (solvent accessibility). The statistically significant association between predicted and measured mRNA ASA values (*P*-value ∼ 0) suggests that the SVM model in RNAsnap is able to capture the sequence–structure relation of RNAs beyond protein-bound RNAs.

The applicability of RNAsnap to all RNAs is further confirmed by using 2.2 million genetic variants found in the 1000 Genomes Project. We showed that mutations of predicted buried RNA bases have deleterious functional impacts as evidenced by their stronger associations with the lower allele frequencies with PCC > 0.8, regardless of whether the mutation site is in coding, 3′ UTR, 5′ UTR, or ncRNA regions. Significant correlations for both RNAsnap-seq and RNAsnap-prof in most cases (except RNAsnap-seq in ncRNA regions), and stronger correlations for more accurate RNAsnap-prof, suggest that the association of MAF with ASA is robust. The lack of correlation in ncRNA regions by RNAsnap-seq suggests that the sequences in noncoding regions have weaker structural signal than in coding, 3′ UTR, and 5′ UTR regions. Moreover, we found that predicted secondary structure has a weaker correlation to MAF than predicted ASA. This finding suggests that mutations in buried RNA regions are more disruptive than mutations in secondary structure regions, confirming the important functional role of RNA tertiary structures.

The above results further illustrate that functional conformations are encoded in RNA sequences even if unbound structures are flexible with dynamic transitions between different structural conformations. In other words, functional structures are within the dynamic structural ensemble and bound structures are likely formed through conformational selection upon binding. This mechanism is increasingly supported by experimental studies (Fürtig et al. 2010; Herschlag et al. 2015).

Success in correlating to in vivo DMS accessibility and allele frequencies of genetic variants in the 1000 Genomes Project encouraged us to make genome-scale application of RNAsnap to 15,642 gene transcripts. We found that introns are the most solvent-exposed whereas exons are the least exposed (Fig. 4). More exposed introns are consistent with the need to interact with the spliceosome for splicing (Hang et al. 2015). The more exposed 5′ cap of 5′ UTR and 3′ cap of 3′ UTR facilitate their binding with regulatory proteins during cotranscriptional splicing and nuclear transportation (Barrett et al. 2012).

While genome-wide DMS accessibility studies of pre-mRNA are not yet available, genome-wide mapping of secondary structures across 5′ UTR, code regions, and 3′ UTR has been made (Wan et al. 2014; Spitale et al. 2015). It was shown that there was a sudden drop and then fast rise in secondary-structure PARS scores near the start codon as well as a sudden drop near stop codons. Predicted solvent accessible surface area (shown in Fig. 4), on the other hand, has larger fluctuation near start and stop codons. This difference simply reflects that secondary structures (paired or not paired) and solvent accessibility (exposed or buried) at the tertiary structural level are inherently different structural properties.

The above multiple independent tests adequately addressed the potential issue of overfitting and the concern of small training and testing data. This is reflected from the fact that the Pearson correlation coefficients for fivefold cross-validation are nearly the same as those for independent tests (0.60 for TR89 and 0.54 for TS44 by RNAsnap-seq and 0.66 for TR89 and 0.63 for TS44 by RNAsnap-prof), although TS44 are made of lower resolution RNA structures with less accurate calculation of experimental accessible surface areas. These correlations found here are considered as moderate (0.4–0.6) or strong (>0.6). Moreover, application of our method to experimentally measured DMS accessibility of >6000 mRNAs reveals weak (PCC = 0.37) but statistically significant correlations (*P*-value ∼ 0) to in vivo DMS accessibility, although DMS accessibility is not the same as ASA for which the method was trained. This correlation is not achieved by accident because only very weak correlation (<0.01) was found between predicted secondary structure and DMA accessibility and only weak correlation (<0.1) between RNAsnap and in vitro DMS accessibility. The ability of our methods to predict the “never seen” data confirms the generality of the method. In addition, although our data set is small in terms of number of RNA structures, it is not small in terms of the number of nucleotides (27216 for TR89 and 8392 for TS44).

In this study, we have established two separate methods. RNAsnap-seq uses RNA sequence as the only input, whereas RNAsnap-prof utilizes the sequence profile generated from multiple sequence alignment. Using RNAsnap-seq is to address the question if a functional structure is somewhat encoded by the sequence alone, in the absence of evolution information and binding partner(s). The answer is affirmative because RNAsnap-seq can make a reasonably accurate prediction of ASA for protein-bound structures with PCC = 0.54 for the independent test set TS44. RNAsnap-prof is used to illustrate if sequence conservation information contained in multiple sequence alignment is useful for more accurate determination of tertiary structural properties. A significant increase in the PCC values between predicted and actual ASAs from 0.54 to 0.63 by using the sequence profile confirmed that an RNA base can be conserved for its tertiary structural role (Capriotti and Marti-Renom 2010; De Leonardis et al. 2015). Sequence conservation in RNA secondary structure (Gruber et al. 2008; Gu et al. 2014) has also been studied and used for secondary structure prediction (Rivas 2013; Sharma et al. 2014; De Leonardis et al. 2015).

It should be noted, however, that if a nucleotide is highly conserved, it could be conserved for its functional or structural role. The use of RNAsnap-seq without sequence conservation information allows us to be certain that the disruption of RNA structure plays a significant role in disease-causing genetic variants based on the correlation between average predicted ASAs and average minor allele frequencies. RNAsnap-prof, on the other hand, can capture both structural and functional disruption caused by disease-causing genetic variants. This explains the small performance difference between RNAsnap-prof and RNAsnap-seq in ASA prediction, but the large difference in function-disruption prediction due to mutation.

We noted that unlike RNA, solvent accessible surface areas of amino acid residues in proteins have long been predicted with high accuracy (Zhou and Faraggi 2010), utilized in applications ranging from protein structure prediction (Karchin et al. 2003; Yang et al. 2011), functional site prediction (Wodak and Mendez 2004; Fischer et al. 2008), and deleterious mutation classifications (Krishnan and Westhead 2003; Zhao et al. 2013). Significant correlation of RNAsnap results to in vivo RNA accessibility indicates that the method will help in improving our qualitative and quantitative understanding of RNA structure and function in vivo beyond the secondary structural level for the first time. In particular, predicted RNA accessibility can serve as a useful restraint for selecting or predicting functional RNA structures. RNAsnap is freely available as a web-based server and a downloadable stand-alone package at http://sparks-lab.org/server/RNAsnap.

## MATERIALS AND METHODS

### Data sets

We built our training (protein-bound) sets based on high-resolution RNA structures. We downloaded 1093 RNA-containing structures with resolution <3.0 Å and RNA chains longer than 32 bases from the Protein Data Bank (March 2015). After removing redundant chains by cd-hit-est (Li and Godzik 2006), with the lowest allowed sequence-identity cutoff at 80%, 137 RNA chains remained. No redundant chains were found by BLASTclust (Altschul et al. 1997) with a cutoff of 30%. To obtain an accurate calculation of solvent accessible surface area, we only include those RNA bases and their two nearest sequence neighbors that do not contain missing atoms. This data set was then divided into those RNA chains in complex with proteins (TR89) and those that are not (CN48), for training (TR) and control (CN), respectively. Because the above data set of high-resolution RNA structures is not large, we decided to build our independent test set by collecting protein-bound RNA structures in lower resolution between 3.0 Å and 4.0 Å. After removing redundancy within itself and to TR89 and CN48, we obtained 44 RNA chains in complex with proteins (TS44) for independent test (TS). RNA structures in TR89, TS44, and CN48 are listed in Supplemental Table S1.

### Sequence profile from multiple sequence alignment

Homologous sequences for each query sequence were searched by BLASTN (Altschul et al. 1997) with *E*-value < 0.001 and a maximum of 50,000 homologous sequences in the nonredundant nucleotide (NT) database. The probability of *j* base (*j* = A, U, G, T/U) in multiple aligned homologous sequences at a given sequence position *i*, *P*_*i,j*_ was calculated by using *P*_*i*__,*j*_ = − log[(*N*_*i,j*_)/∑_j_(*N*_*i*__,*j*_)], where *N*_*i,j*_ is the observed number of base type *j* at position *i.* To avoid zero occurrences, a small number correction *s(b_i_)* was added to *N*_*i,j*_ based on the normalized expected average occurrence for the native base type and other types. *s(b_i_)* is set to 9.0 for the query base type and 0.3 for the other base type *b*_*i*_. The sequence profile was normalized to a range of (−1,1) before training or test.

### Training

Residue-specific ASA for each RNA chain was calculated with a solvent probe diameter of 1.5 Å by the TINKER package (Ponder and Richards 1987). All ASA values were normalized uniformly by 400 Å^2^ because there is no significant difference between the largest accessible areas for different base types. Two separate prediction methods were developed. In the single-sequence-based method (RNAsnap-seq), a sequence of length *L* was defined by a 4 × *L* matrix where each sequence position was represented by a four-dimensional vector with 1 for the query base type at the position and −1 for other base types. In the sequence-profile-based technique (RNAsnap-prof), this four-dimensional vector was replaced by the profile generated from multiple sequence alignment described above. These sequence-based features within a neighboring window *w* around the query sequence position were utilized as input; that is, the number of features is 4×(2 × *w* + 1). A vector of (−1,−1,−1,−1) was used for missing neighboring bases at the terminal ends. We used support vector machines (SVMs) implemented in libsvm version 3.12 (Chang and Lin 2011) for predicting real values of ASAs based on input features. We optimized the window size with default libsvm parameters for the best performance in the fivefold cross-validation, and reoptimized SVM parameters (c and γ) with the optimal window size (*w* = 40). The fivefold cross-validation was done by randomly dividing RNA chains in TR89 into five parts and each part (fold) was used in turn as a test by using the remaining four folds for training. The dependence of Pearson's correlation coefficient on the window size is shown in Supplemental Figure S8. Only sequence-based features were used here to minimize the possibility of overtraining. The final method was tested by the protein-free control (CN48) and the independent test set (TS44).

### DMS data

DMS reactivity data for all mRNAs of human bone marrow K562 cells was downloaded from NCBI (geo: GSE45803). We obtained 6178 mRNA chains with at least 4000 reacted bases at four experimental conditions: denature, in vitro, vivo300 (in vivo treated with 300 µL DMS), and vivo400 (in vivo treated with 400 µL DMS) (Rouskin et al. 2014). In this study, only results on vivo300 data were reported because vivo400 data gave essentially the same correlation to our predicted ASA. We normalized the reads by the maximum read on each mRNA to remove the effect of mRNA abundance. The normalized reads were averaged over all nonoverlapping 50-base fragments, and the fragments with null counts in the denatured state or the to-be-compared state were removed. To further remove potential systematic bias by the location of bases, in vitro and in vivo data were also normalized by denatured results.

### 1000 Genomes data

We downloaded the 1000 Genomes Phase 3 VCF file from Ensembl annotated by ANNOVAR (Wang et al. 2010). We obtained single-nucleotide variations (SNVs) along with their minor allele frequencies (MAF) occurring in 5′ untranslated region (UTR, 165,055 cases), 3′ UTR (734,800 cases), noncoding RNA (ncRNA, 255,118 cases), and coding regions (616,192 nonsynonymous and 384,893 synonymous cases). MAF is the frequency of the least common allele in a population. For each category, SNVs were sorted and equally separated into 100 bins according to predicted ASAs (or unpaired probability for secondary structure) at their mutation positions. Predicted values and log(MAF) were averaged and Pearson's correlation coefficients were calculated based on the average values.

### Human genome data

We downloaded all 21,983 protein-coding gene sequences in the human genome from ENSEMBL version 83 (Hubbard et al. 2002). After removing the genes without transcripts containing 5′ UTR, coding DNA sequence (CDS), and 3′ UTR regions, and the genes with less than 100 bases for any region, 15,642 genes remained. For convenience we chose the transcript with the longest CDS region to represent each gene. The statistics in Figure 4 were obtained by averaging ASA results at 50 bases upstream and downstream around each boundary line.

### RNAplfold

For genome-scale studies, we compared our RNAsnap to secondary-structure prediction software RNAplfold from the package Vienna RNA 2.1.9 (Lorenz et al. 2011). Default parameters were utilized.

## SUPPLEMENTAL MATERIAL

Supplemental material is available for this article.

## Supplementary Material

Supplemental Material
