# Supplementary material for: Genome-scale characterization of RNA tertiary structures and their functional impact by RNA solvent accessibility prediction
Source: RNA. 2017 Jan;23(1):14–22. doi: 10.1261/rna.057364.116 (PMC5159645; doi:10.1261/rna.057364.116)
Supplement: Supplemental Material [file supp_23_1_14__index.html]

Genome-scale characterization of RNA tertiary structures and their functional impact by RNA solvent accessibility prediction — Supplemental Material 

# Genome-scale characterization of RNA tertiary structures and their functional impact by RNA solvent accessibility prediction

## Supplemental Material

**Files in this Data Supplement:**

- Supplemental Material.pdf
