# Supplementary material for: Genome-scale characterization of RNA tertiary structures and their functional impact by RNA solvent accessibility prediction
Source: RNA. 2017 Jan;23(1):14–22. doi: 10.1261/rna.057364.116 (PMC5159645; doi:10.1261/rna.057364.116)
Supplement: Supplemental Material [file supp_057364.116_Supplemental_Material.pdf]

Supplementary Materials: Genome-scale characterization of RNA tertiary structures and their functional impact by RNA solvent accessibility prediction

Yuedong Yang[1], Xiaomei Li[1,2], Huiying Zhao[3], Jian Zhan[1], Jihua Wang[4] and Yaoqi Zhou[1,4]*

[1]Institute for Glycomics and School of Information and Communication Technology, Griffith University, Gold Coast, QLD 4222, Australia.

[2]School of Computer Science and Information Engineering, Hefei University of Technology, Hefei 230009, PR China

[3]Institute of Health and Biomedical Innovation, Queensland University of Technology, Queensland, Australia

[4]Shandong Provincial Key Laboratory of Biophysics, Institute of Biophysics, Dezhou University, Dezhou 253023, China

*To whom correspondence should be addressed (Institute for Glycomics and School of Information and Communication Technology, Griffith University, Gold Coast, QLD 4222, Australia; 61-755528228; yaoqi.zhou@griffith.edu.au)

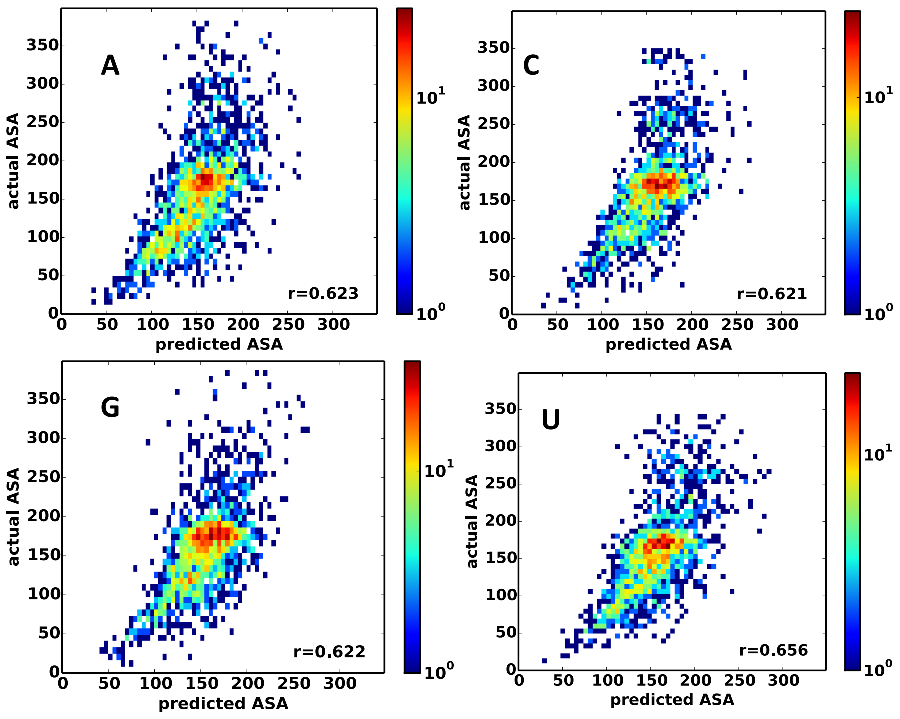

Figure S1. **Performance on the independent test (TS44) set of protein-bound structures by RNAsnap.** The density plot of predicted versus actual ASA values on TS44 by RNAsnap-prof for each individual base as labelled. PCC values (*r*) are as labelled.

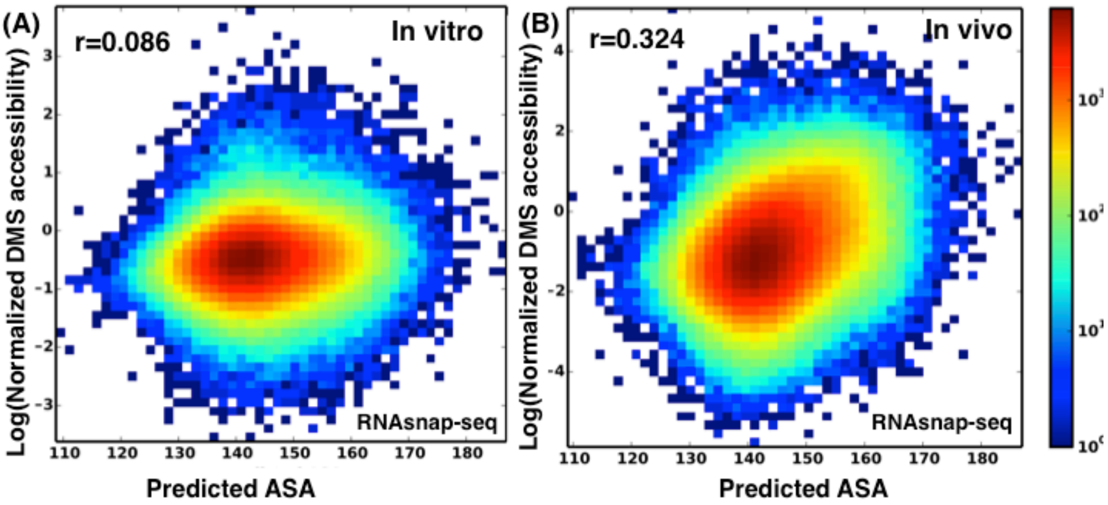

Figure S2. **RNAsnap-seq prediction correlates to *in vivo* but not to *in vitro* data**. The density plot of normalized *in vitro* (A) and *in vivo* (B) DMS accessibilities versus predicted ASA values by RNAsnap-seq. The Pearson's correlation coefficient r=0.086 and 0.324 for *in vitro* and *in vivo* data, respectively.

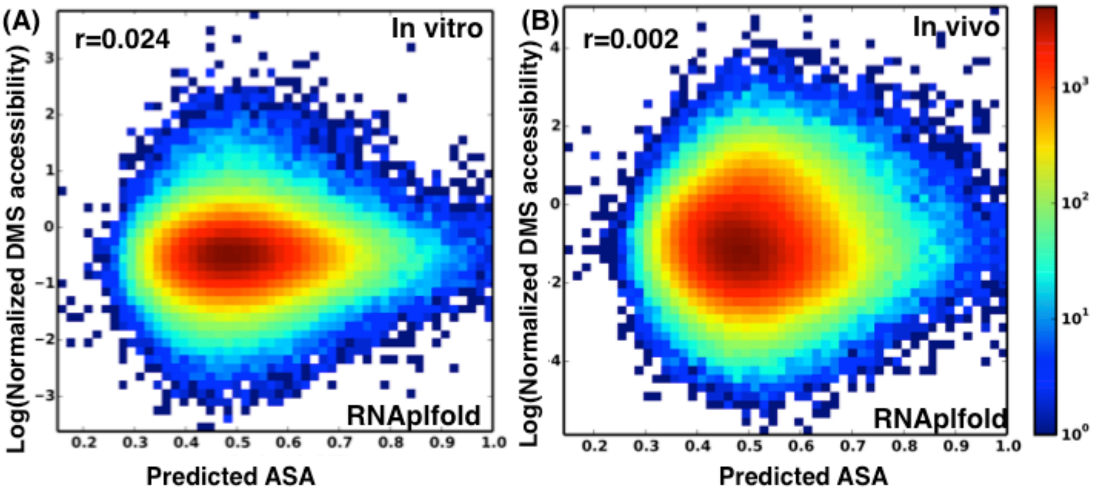

Figure S3. As in Figure S1 but for RNAplfold.

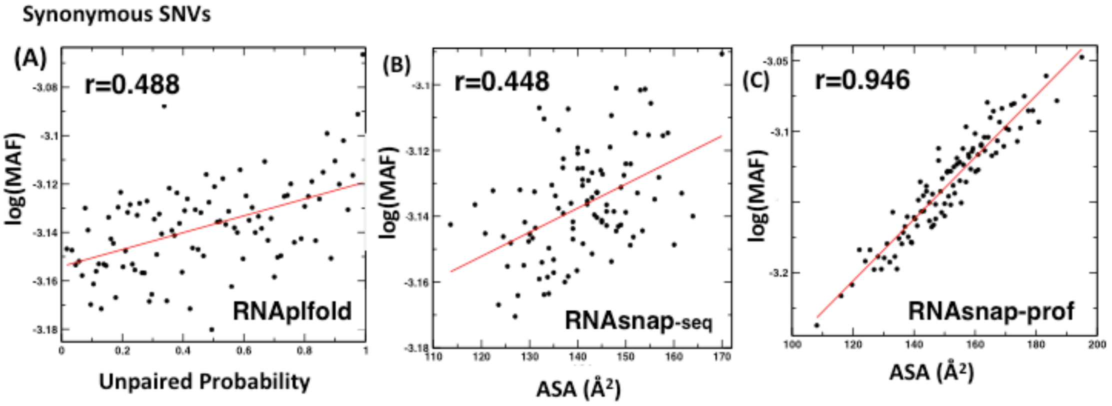

Figure S4: **Positive association between MAF of genetic variants and predicted ASA or secondary structures in** <u>Synonymous SNVs</u>**.** The average MAF of single nucleotide variations from the 1000 genomes project versus unpaired probability by the secondary structure predictor RNAplfold (A), predicted ASA by RNAsnap-seq (B), and RNAsnap-prof (C). Pearson's correlation coefficient *r* is as labelled.

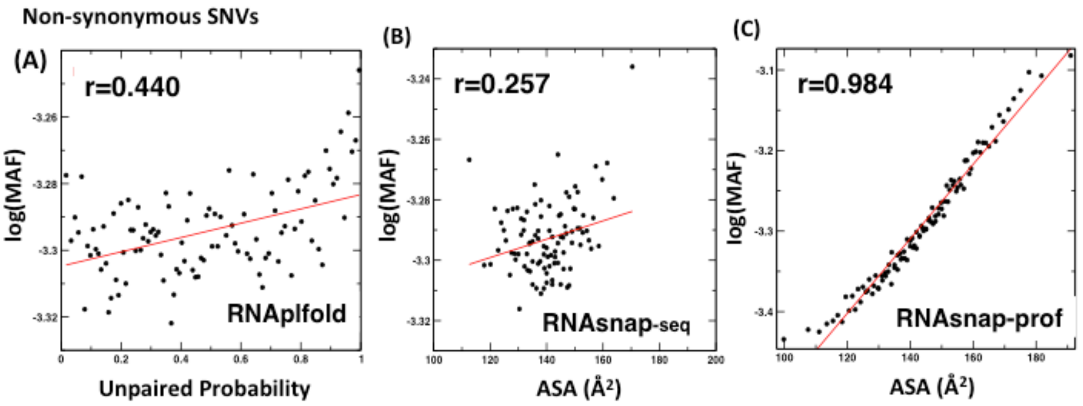

Figure S5. As in Figure S3 but for <u>non-synonymous SNVs</u>**.**

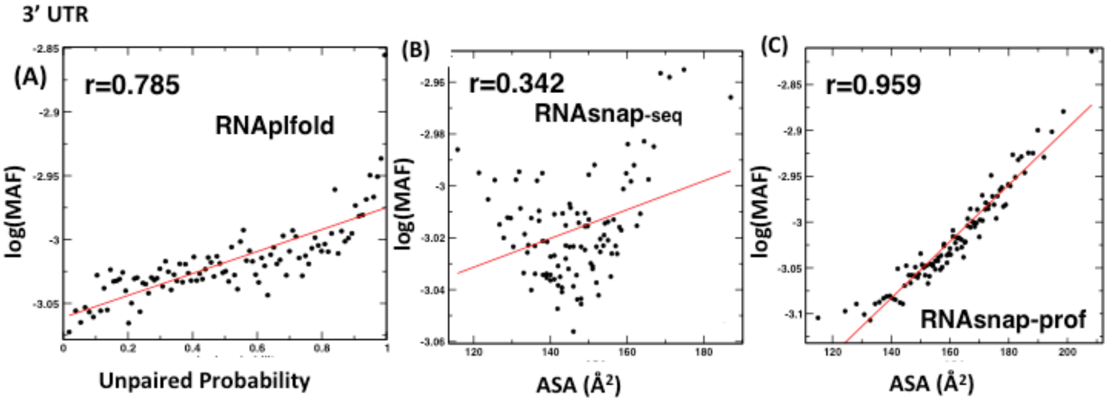

Figure S6. As in Figure S3 but for <u>3' UTR</u>**.**

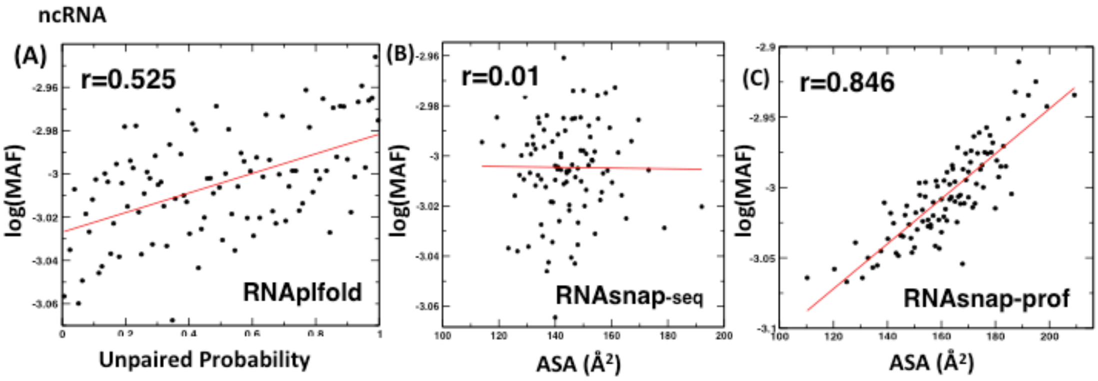

Figure S7. As in Figure S3 but for non-coding RNA.

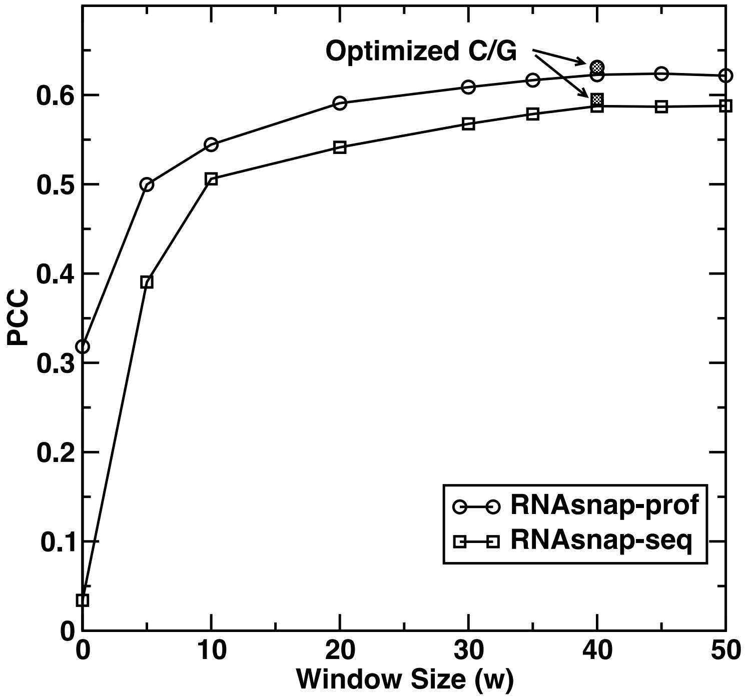

Figure S8. Pearson Correlation Coefficient (PCC) of five-fold cross-validation as a function of window size *w* (TR89).

Table S1. Lists of RNA chains for training set TR89, independent test set TS44, and control set TN48

TR89	4u3m_1 4v9o_AA 4lnt_RA 4v51_BA 3cc2_0 2zjr_X 4u3m_2 4v9o_BA 4v67_AA 2r8s_R 4u3m_4 3ndb_M 3hhn_C 2nz4_P 3v7e_C 1l9a_B 2zjr_Y 1ffk_9 4u3m_3 4v67_BB 4v9o_AB 3adb_C 1m5k_B 3irw_R 3cul_C 4v8b_AB 2zzm_B 4kzd_R 4v8d_AB 3am1_B 3hl2_E 3amt_B 4v90_AV 2zue_B 2csx_C 1gax_C 1g59_B 1ffy_T 4rdx_C 3zgz_B 3akz_F 1j1u_B 1sj3_R 2azx_C 2zzn_C 2du3_D 3eph_E 1h3e_B 1vy5_AX 3kfu_K 4n0t_B 1h4q_T 3egz_B 4u7u_L 3rw6_H 4m4o_B 1qf6_B 3hjw_D 1dk1_B 1b23_R 4pkd_V 1c0a_B 1ser_T 1asy_R 2hvy_E 2fk6_R 4c7o_E 2vpl_B 1dul_B 1s03_A 3iab_R 2nue_C 4o26_E 1ttt_D 1g1x_D 4kr6_C 4pdb_I 2dlc_Y 1zho_B 1i6u_C 4x4p_B 3vjr_B 3ouy_C 2xdb_G 4oog_D 3nmu_D 2zh2_B 4ato_G 2ozb_C
TS44	4v8p_A1 4bts_AA 3q1q_B 1u6b_B 3p49_A 4v8p_B2 4uyk_R 4v8p_B3 4lck_C 2xxa_F 4w90_C 2gtt_X 2gtt_W 3w3s_B 3ktw_C 3k0j_E 3q1q_C 4xjn_N 4v8n_AW 4v9j_AW 3a2k_C 1j2b_C 4wj3_Q 4v9i_AY 4lck_B 3wqy_C 3tup_T 2der_C 2zni_C 2d6f_E 2yhm_K 4wzj_V 4kr2_C 4v8q_BY 1ob5_B 1un6_E 4tvx_L 2czj_B 4pjo_1 3ciy_C 1p6v_B 4pmi_A 1kog_I 3pla_G
CN48	3g78_A 4p95_A 3dig_X 1u9s_A 2qbz_X 2gcs_B 4wfl_A 3f2q_X 4qlm_A 3suh_X 4l81_A 2gis_A 3sd3_A 3owi_A 4frg_B 4p5j_A 2cky_A 4jf2_A 3q3z_V 2oiu_P 1y26_X 1kxk_A 4pqv_A 2qus_A 1y27_X 3ski_A 2hoj_A 4jrc_A 4k27_U 4rge_A 3vrs_A 3e5c_A 2qwy_A 4oji_A 1yfg_A 3npn_A 1xjr_A 2oeu_A 3p22_A 4ts0_X 4fnj_A 1ddy_A 3gs5_C 1kh6_A 3gca_A 3fu2_A 1yls_B 1f1t_A
